# Promotion of couples’ voluntary HIV counseling and testing: a comparison of influence networks in Rwanda and Zambia

**DOI:** 10.1186/s12889-016-3424-z

**Published:** 2016-08-08

**Authors:** April L. Kelley, Ashley K. Hagaman, Kristin M. Wall, Etienne Karita, William Kilembe, Roger Bayingana, Amanda Tichacek, Michele Kautzman, Susan A. Allen

**Affiliations:** 1 Rwanda Zambia HIV Research Group (RZHRG), Department of Pathology & Laboratory Medicine, School of Medicine, Emory University, Atlanta, GA USA; 2 Hubert Department of Global Health, Rollins School of Public Health, Emory University, Atlanta, GA USA; 3 Project San Francisco (PSF), Kigali, Rwanda; 4 Zambia Emory HIV Research Project (ZEHRP), Lusaka and Ndola, Zambia; 5 Department of Epidemiology, School of Public Health, Emory University, 1518 Clifton Road NE, GCR 458, Atlanta, GA 30322 USA

**Keywords:** HIV/AIDS, Couples’ voluntary HIV counseling and testing, HIV education, Health service promotion, Influential networks

## Abstract

**Background:**

Many African adults do not know that partners in steady or cohabiting relationships can have different HIV test results. Despite WHO recommendations for couples’ voluntary counseling and testing (CVCT), fewer than 10 % of couples have been jointly tested and counseled. We examine the roles and interactions of influential network leaders (INLs) and influential network agents (INAs) in promoting CVCT in Kigali, Rwanda and Lusaka, Zambia.

**Methods:**

INLs were identified in the faith-based, non-governmental, private, and health sectors. Each INL recruited and mentored several INAs who promoted CVCT. INLs and INAs were interviewed about demographic characteristics, promotional efforts, and working relationships. We also surveyed CVCT clients about sources of CVCT information.

**Results:**

In Zambia, 53 INAs and 31 INLs were surveyed. In Rwanda, 33 INAs and 27 INLs were surveyed. Most (75 %–90 %) INAs believed that INL support was necessary for their promotional work. Zambian INLs reported being more engaged with their INAs than Rwandan INLs, with 58 % of Zambian INLs reporting that they gave a lot of support to their INAs versus 39 % in Rwanda. INAs in both Rwanda and Zambia reported promoting CVCT via group forums (77 %–97 %) and speaking to a community leader about CVCT (79 %–88 %) in the past month. More Rwandan INAs and INLs reported previous joint or individual HIV testing compared with their Zambian counterparts, of which more than half had not been tested. In Zambia and Rwanda, 1271 and 3895 CVCT clients were surveyed, respectively. Hearing about CVCT from INAs during one-on-one promotions was the most frequent source of information reported by clients in Zambia (71 %). In contrast, Rwandan couples who tested were more likely to have heard about CVCT from a previously tested couple (59 %).

**Conclusions:**

CVCT has long been endorsed for HIV prevention but few couples have been reached. Influential social networks can successfully promote evidence-based HIV prevention in Africa. Support from more senior INLs and group presentations leveraged INAs’ one-on-one promotions. The INL/INA model was effective in promoting couples to seek joint HIV testing and counseling and may have broader application to other sub-Saharan African countries to sustainably increase CVCT uptake.

## Background

Sub-Saharan Africa, where an estimated 1.8 million people were newly HIV infected and 1.2 million died of HIV-related causes in 2011 [1], continues to be disproportionately affected by HIV/AIDS. Heterosexual transmission accounts for the majority of HIV infections in sub-Saharan Africa [2], where 35 % to 80 % of all heterosexual HIV transmissions occur in stable relationships [3]. Voluntary HIV counseling and testing (VCT) programs are an effective and feasible approach for increasing serostatus awareness and decreasing high-risk behavior [4]. Couples’ voluntary HIV counseling and testing (CVCT), in which both partners test, share their results and formulate risk-reduction plans based on their couple-level serostatus, facilitates disclosure of sensitive information within relationships and effectively targets couples, one of the most critical prevention points in sub-Saharan Africa [5–10].

Though CVCT is an effective prevention service, it has not yet been widely implemented. Measures are needed to increase communities’ access to HIV prevention programs, combat stigma, and increase utilization of CVCT services. Several studies have shown that influential social networks and community leaders can change attitudes and risk perceptions towards HIV/AIDS and link individuals to health services in Africa [11–16].

The Rwanda Zambia HIV Research Group (RZHRG), consisting of Project San Francisco (PSF) in Kigali, Rwanda and the Zambia Emory HIV Research Project (ZEHRP) in Lusaka, Zambia, provides CVCT services. Initially, RZHRG promotions relied on influential network agents (INAs) to distribute invitations for CVCT and provide information about the service within the communities; after a period of pilot testing of this promotional system, these INAs suggested that they would benefit from the support of even more influential members of the community [17]. This suggestion led to the incorporation of influential network leaders (INLs) into the promotional model, with INAs and INLs each serving a distinct advocacy role. INLs instilled a shared vision in the community in the fight against HIV, nominated INAs, publicly promoted CVCT, and supported INAs in their CVCT promotional work. INAs interacted directly with couples in the community by giving information and written invitations for couples to seek CVCT.

The purpose of this study was to describe promotional efforts and relationships between INAs and INLs in Kigali and Lusaka to better understand the methods and styles of their work, and to describe where CVCT clients were receiving information about CVCT. The information from this study aimed to determine the most effective methods of disseminating information about CVCT in the community to increase the number of couples seeking joint HIV counseling and testing.

## Methods

### Recruitment and data collection: INAs and INLs

INAs and INLs were identified by RZHRG staff and trained to promote CVCT. Detailed INA and INL recruitment, training and follow-up methods have been previously described [17–22]. Briefly, RZHRG staff identified INLs from consensus meetings and national/citywide umbrella referrals from four social networks (faith-based/religious, health, private and community-based/non-governmental organizations (CBOs/NGOs)). INLs identified INA candidates from their respective networks, and RZHRG counselors made a final selection after interviewing. After completing IRB-approved written informed consents and demographic questionnaires, enrolled INAs received 4-day training in HIV/AIDS health advocacy/outreach, social networking, CVCT promotions and observation of successful door-to-door promotional strategies. Testing for HIV alone or with partners was offered but not required as part of INL and INA training. Weekly follow-up meetings were held to discuss challenges INAs encountered during their work. Questionnaires assessing INA and INL relationships were developed collaboratively by the first author, the project directors at Emory and at each field site, and in-country staff. Questionnaire content was based on findings from focus groups. Topics covered in the questionnaires included socio-demographic information; questions regarding promotional efforts by INAs and INLs, including time spent promoting CVCT, the number of group endorsements given, the size of group endorsement sessions, and engagement with community leaders; and the type of audience INAs and INLs promoted to, including couples, women alone, men alone, or mixed gender groups. The questionnaires also ascertained the working relationship between the INLs and INAs, how often the INAs and INLs met and for how long, if INAs believed INLs were necessary to their promotional work, and the perceived level of support INLs gave to their INAs.

Senior counselors and INA trainers, all of whom were Rwandan or Zambian and familiar with the culture and local language, reviewed the questionnaires for content and cultural appropriateness. Additionally, senior counselors piloted the survey with four INAs and four INLs in each city. The final INA and INL questionnaires contained 31 and 29 items, respectively. Answer formats included Yes/No, multiple choice, and open-ended questions. The instruments were translated and back-translated by RZHRG counselors. One-on-one administration of the questionnaires lasted between 20 and 30 min. INAs were reimbursed $4 each for their time and transport, and INLs were reimbursed $6 each for their time and transport.

### Recruitment and data collection: CVCT couples

CVCT procedures have been previously described [17, 19, 20, 22]. Couples presented to RZHRG testing sites and participated in a group counseling session, followed by a confidential pre-test joint session. Afterwards, the couple could elect to continue and be HIV tested. Those that were tested received joint post-test counseling. A standard questionnaire was administered to all incoming couples to determine where they had heard of CVCT, including community members and mass media sources that provided information about CVCT. INL and INA interviews were conducted by local nurses in either French/Kinyarwanda (in Kigali) or English/Nyanja (in Lusaka).

### Data analysis

INA, INL, and couple-level variables were described by country using counts and percentages for categorical variables and means and standard deviations for continuous variables. Bivariate relationships between Rwandan and Zambian INAs and INLs were examined using independent two-sample *t*-tests or non-parametric tests for differences between means and Chi-square or Fisher’s Exact tests for differences between frequency distributions, as appropriate. When the equality of variances (F) *p*-value was less than 0.05, Satterthwaite*t*-test statistics and *p*-values were reported. All reported *p*-values are two-tailed. Data were analyzed using Statistical Analysis Software (SAS) v9.3 (Cary, NC).

## Results

At the time of the study, Lusaka employed 65 INAs and 37 INLs: 53 and 31, respectively, completed their contracts during the period of the study and were available for exit interviews. Similarly, Kigali sites employed 63 INAs and 38 INLs, and 33 and 27 completed the study, respectively.

### INA and INL demographics (Table 1)

**Table 1:** Demographic characteristics of influential network agents and leaders by country

	INAs		INLs		*P* value
	Rwanda % (n/N) or mean (SD)	Zambia % (n/N) or mean (SD)	Rwanda % (n/N) or mean (SD)	Zambia % (n/N) or mean (SD)	
Gender					n/s
Woman	64 (21/33)	55 (29/53)	78 (21/27)	71 (22/31)	
Man	36 (12/33)	45 (24/53)	22 (6/27)	29 (9/31)	
Age	33 (7)	39 (10)	40 (9)	46 (9)	*, **, ***, ****
Ever married	82 (27/33)	82 (42/52)	89 (24/27)	93 (27/29)	n/s
Job Type					n/s
Professional/sales/service	64 (21/33)	67 (8/12)	86 (24/28)	81 (13/16)	
Unskilled manual/technical/other	36 (12/33)	33 (4/12)	14 (4/28)	19 (3/16)	
Ever tested for HIV					***
Yes, alone	47 (15/32)	20 (10/50)	25 (7/28)	23 (7/30)	
Yes, with spouse	31 (10/32)	24 (12/50)	43 (12/28)	23 (7/30)	
No testing reported	22 (7/32)	56 (28/50)	32 (9/28)	53 (16/30)	

*Significant (*p* < 0.05) differences between INAs and INLs in Rwanda

**Significant (*p* < 0.05) differences between INAs and INLs in Zambia

***Significant (*p* < 0.05) differences between Rwandan and Zambian INAs

****Significant (*p* < 0.05) differences between Rwandan and Zambian INLs

Note: denominators are variable due to missing data

Demographic characteristics of INAs and INLs in Rwanda and Zambia are presented in Table 1. Both Rwandan and Zambian INAs and INLs tended to be women who were currently or previously married and employed in a professional sales or service capacity. Key differences between Rwandan and Zambian INAs and INLs included age and having had a previous HIV test. Zambian INAs and INLs were an average of six years older than their Rwandan counterparts. As expected, given their more senior status, INLs were an average of seven years older than INAs in both cities. More than half of Zambian INAs and INLs did not report prior HIV testing, and half of those who had tested had done so alone. In contrast, most Rwandan INAs had tested alone or with their spouse, while Rwandan INLs were the most likely to have tested with their spouse.

### INA and INL promotional work (Table 2)

**Table 2:** CVCT promotional efforts by influential network agents and leaders by country

	INAs		INLs		*P* value
	Rwanda % (n/N) or mean (SD)	Zambia % (n/N) or mean (SD)	Rwanda % (n/N) or mean (SD)	Zambia % (n/N) or mean (SD)	
Number hours per week promoting CVCT	28 (18)	18 (9)	5 (5)	10 (6)	*, **, ***, ****
Gave endorsement to group in past month	97 (32/33)	77 (41/53)	96 (27/28)	100 (31/31)	**, ***
Number of group endorsements in past month	2 (1)	4 (5)	6 (7)	10 (18)	*, ***
Number of attendees to group endorsements	38 (33)	55 (45)	101 (194)	102 (107)	**
Number of INAs present at public endorsements	*n/a*	*n/a*	4 (5)	8 (10)	****
Spoke to community leader in past month	88 (29/33)	79 (42/53)	89 (25/28)	81 (25/31)	n/s
Number of community leaders spoken to in past month	4 (3)	5 (5)	10 (13)	14 (13)	*, **
Public endorsement audience					*, ***, ****
All public endorsement audiences	3 (1/30)	43 (17/40)	37 (10/27)	61 (19/31)	
Couples	63 (19/30)	10 (4/40)	26 (7/27)	3 (1/31)	
Married women	13 (4/30)	40 (16/40)	11 (3/27)	32 (10/31)	
Married men	3 (1/30)	5 (2/40)	0 (0/27)	3 (1/31)	
Men and Women (Not in relationships)	17 (5/30)	3 (1/40)	26 (7/27)	0 (0/31)	

*Significant (*p* < 0.05) differences between INAs and INLs in Rwanda

**Significant (*p* < 0.05) differences between INAs and INLs in Zambia

***Significant (*p* < 0.05) differences between Rwandan and Zambian INAs

****Significant (*p* < 0.05) differences between Rwandan and Zambian INLs

Note: denominators are variable due to missing data

In both cities, INAs spent more hours/week than INLs promoting CVCT, while INLs were more likely to have reached large audiences through multiple large group meetings. INLs had also spoken with more community leaders than INAs. Zambian INLs reported more INAs present at their group endorsements than Rwanda INLs.

Zambian INLs spent more time on CVCT promotion than their Rwandan counterparts, whereas Rwandan INAs spent more time promoting CVCT than Zambian INAs. While approximately equal numbers of Rwandan INAs and INLs (97 and 96 %, respectively) and 100 % of Zambian INLs reported giving public endorsements, only 77 % of Zambian INAs reported public endorsements. On average, however, Zambian INAs gave significantly more public endorsements than their Rwandan counterparts.

In terms of the location and target audience of CVCT promotion, Zambian INAs were more likely than Rwandan INAs to promote CVCT to a group at a church (71 % vs. 44 %, *p* = 0.01) while Rwandan INAs were more likely to promote at an NGO or CBO in the community (38 % vs. 7 %, *p* < 0.001) (data not shown). Zambian INAs were more likely than INLs to promote CVCT in a church venue (71 % vs. 48 %, *p* = 0.04), whereas INLs were more likely than INAs to promote in a school setting (32 % vs. 10 %, *p* = 0.01) (data not shown). Rwandan INAs and INLs reported speaking most often to groups of couples or men and women together, while Zambian INAs and INLs most often spoke to married women or a variety of groups.

### INA and INL working relationships (Table 3)

**Table 3:** Working relationships between influential network agents and leaders by country

	Rwanda		Zambia	
Self-reported by INA	n/N	%	n/N	%
Relationship with INL				
Superior/Boss	7/30	23	17/53	32
Co-worker	11/30	37	14/53	26
Advisor	7/30	23	6/53	11
Religious Group Member	1/30	3	7/53	13
Friend	3/30	10	5/53	9
Relative	1/30	3	2/53	4
No relationship	0/30	0	2/53	4
Met with INL in past month	29/32	91	43/53	81
Number of times INA met with INL in past month, mean (SD)	7	(8)	7	(7)
Number of minutes INA and INL met, mean (SD)	32	(19)	45	(37)
Help received from INL				
A lot	11/31	35	22/53	42
Some	9/31	29	11/53	21
A little	6/31	19	9/53	17
None	5/31	16	11/53	21
Believe INL is necessary to their work	28/31	90	40/53	75
Self-reported by INL				
Met with INA in past month	25/28	89	30/31	97
Number of times INL met with INA in past month	6	(5)	8	(7)
Number of minutes INA and INL met*	29	(21)	50	(44)
Number of INAs worked with*	2	(1)	3	(2)
Help given to INA				
A lot	7/18	39	18/31	58
Some	9/18	50	12/31	39
A little	1/18	6	1/31	3
None	1/18	6	0/31	0

*p < 0.05

Table 3 depicts the working relationships between INAs and INLs in Kigali and Lusaka. Each INL reported mentoring and supporting an average of 2 (Kigali) to 3 (Lusaka) INAs (*p* < 0.05). INAs in both cities reported that INLs were most often their superiors, co-workers or advisors. More Zambian than Rwandan INAs described INLs as fellow religious group members. In both cities, a minority of INAs reported being friends or family members of their INLs.

INAs in both cities met with their INLs an average seven times/month for an average of 32-45 min. Zambian INLs reported significantly longer meetings with INAs relative to Rwandan INLs. While nearly all of the INLs believed they were very or somewhat helpful, only two-thirds of INAs reported that INLs were very or somewhat helpful to the INAs promotional efforts.

### CVCT client sources of CVCT information (Table 4)

**Table 4:** CVCT Clients’ sources of information about CVCT

	Rwanda (*n* = 3895)			Zambia (*n* = 1271)		
	In a group	One-on-one	Had not heard	In a group	One-on-one	Had not heard
Heard about CVCT from (community members)...	%	%	%	%	%	%
INA	2.3	35.8	61.9	0.5	71.0	28.5
INL	0.1	0.8	99.2	0.2	3.4	96.5
Other ZEHRP staff	0.03	3.6	96.4	0.1	2.1	97.8
Friends	0.2	12.2	87.7	0.2	15.3	84.5
Family	0.03	4.5	95.5	0.0	6.1	93.9
Couple previously tested at ZEHRP	0.1	59.4	40.5	0.1	12.0	88.0
Other	0.1	16.1	83.8	1.8	8.8	89.4
Heard about CVCT from (media)…	%			%		
Radio	52.8			26.8		
Television	3.7			18.0		
Poster	33.0			3.5		
Newspaper	2.1			3.3		
ZEHRP Brochure	1.2			4.9		
Other brochure	0.2			1.4		
Other	6.6			9.8		

Percentages may not equal 100 due to rounding

Among couples seeking CVCT, very few reported having heard about the services in a group setting, whether from INAs, INLs or others. Twice as many Zambian couples had heard about CVCT one-on-one from an INA, while Rwandan couples were five times more likely to have heard about CVCT from a previously tested couple. Friends and family contributed to 17 % of Rwandan CVCT seekers and 21 % of their Zambian counterparts. Mass media sources were also quite different in the two cities. Radio was the most commonly cited media source of information in both cities, however it was twice as common among Rwandan than Zambian clients. Television was the second most common source in Zambia compared to posters in Rwanda.

## Discussion

CVCT has not been widely implemented and many Africans do not know that two partners in a couple can have different HIV test results [23, 24]. Influential people within social networks can be helpful in promoting health-seeking behaviors in Africa and can raise awareness of the importance of joint HIV testing for couples [17, 19, 22]. This study found that INAs and INLs in both Rwanda and Zambia were working together to promote CVCT to groups and were speaking to other leaders in the Kigali and Lusaka communities about CVCT. Overall, relationships between INAs and INLs in both countries were similar. However, certain INA and INL demographics, promotional activities, and client sources of CVCT differed in Rwanda and Zambia, which may have contributed to the larger number of couples tested in Rwanda compared with Zambia [19, 22]. These differences suggest elements that should be considered when adapting this model to other African contexts.

The finding that Zambian INAs were significantly less likely to have ever tested for HIV (either alone or with a spouse) relative to Rwandan INAs has important implications for CVCT uptake in Zambia where previous studies showed that INAs who had previously tested for HIV with a partner or alone were more successful at promoting CVCT [19]. Recruitment of INAs who have previously tested for HIV or who are willing to test may be more productive if they can speak from a more informed perspective about the process and advantages, particularly if they have tested with a partner.

INAs in both Rwanda and Zambia reported promoting CVCT via group forums. This demonstrated that INAs have the opportunity and ability to speak to groups within their sphere of influence. A prior group of INAs who were not supported by INLs reported lacking confidence and feeling discomfort giving group talks in the community due to stigma and lack of support [17]. This finding suggests an influence of INLs on INAs’ confidence and subsequent promotion to groups. Rwandan INAs, however, reported spending more time promoting CVCT per week than Zambian INAs. Rwandan INAs also reported giving endorsements to groups in the prior month more frequently than Zambian INAs. Increased support and training focused on increasing INA comfort with group CVCT promotions may be useful when adapting this model in other settings.

Audiences of INA and INL endorsements of CVCT were more likely to be comprised of couples or a mixture of all audience types in Rwanda than in Zambia. In Zambia, audiences were more likely to be married women or a mixture of all audience types. Previous studies in Rwanda and Zambia have shown that promotions are more successful when CVCT invitations are delivered to couples versus individuals [19, 22]. Given that communication barriers are often reported by women when discussing and negotiating HIV related issues with their partners [25–27], it is possible that comparatively low testing rates observed in Lusaka in comparison to Kigali, where we have observed CVCT invitation uptake rates of 6 % and 18 %, respectively [19, 22],may be due to insufficient CVCT promotions to married men and couples. Gender power dynamics may be barriers for women to persuade their partner to test regardless of effective INA or INL promotions. Additionally, Rwandan INLs were more likely to promote CVCT to pre-marital and boyfriend/girlfriend partners than Zambian INLs. Promotion to this group is important to increase serostatus awareness among couples who are pre-sexual or considering marriage.

Zambian INAs were more likely than Rwandan INAs to attend public endorsements given by INLs. Previous research conducted by RZHRG found that public endorsements preceding CVCT invitations were a predictor of successful invitations [17, 19]. Though public speaking is an important component of promoter training for both INLs and INAs, INLs by virtue of their more senior status in the community are more likely to be comfortable addressing groups of people.

Most INAs and INLs in Rwanda and Zambia reported speaking to community leaders. This indicates an ability to use their influence in the community to garner the support of other respected community figures for the promotion of CVCT. INLs reported speaking to a greater number of community leaders than INAs, suggesting that positions held by INLs allowed them more access to other and more senior influential leaders in the community. Rwandan and Zambian INAs reported receiving similar amounts of assistance from their INLs. In addition, the majority of INAs in both countries reported that they received help from their INLs and believed that INLs were necessary to their promotional activities. This further strengthens the rationale for a two-tiered promotional program.

Venue may have also impacted the success of promotional activities. Although there was no significant difference between Rwandan and Zambian INLs by public endorsement location, more Zambian INAs reported church as a major location of group talks, reflecting the importance of religion in Zambia. However, the use of religious organizations as venues for promotion of CVCT may be ineffectual if the religious community is sending mixed messages about HIV testing for couples, as was observed during condom campaigns in Mozambique [28]. Rwandan INAs were more likely to promote at an NGO/CBO relative to Zambian INAs. Given that previous studies in Rwanda and Zambia demonstrated that the most successful invitations were delivered in the home [17, 19, 22], it may be beneficial to increase training and support of INAs in home-based CVCT promotions.

INAs and INLs in both Rwanda and Zambia reported meeting with each other often to collaborate on best practices of CVCT promotion. Zambian INAs and INLs both reported a longer duration of meetings than their Rwandan counterparts, possibly attributable to problems during promotion of CVCT in the community that necessitated more time for strategizing and problem solving. Further exploration is needed to better understand how agents and leaders addressed challenges and successes.

Most CVCT clients received information about CVCT via one-on-one sessions versus group sessions. Hearing about CVCT from INAs during one-on-one promotions was the most frequently reported source of information for clients in Zambia. This again suggests the importance of promotional activities in more personal ways and potentially in more discrete locations like the home. In contrast, Rwandan couples who tested were more likely to have heard about CVCT from a previously tested couple. This may reflect the longer history of CVCT in Rwanda [9, 29, 30]. It might also result from the substantially lower HIV-prevalence in Rwandan couples, since concordant HIV-negative couples may be more open about having been tested than couples in which one or both partners HIV positive.

Another potential contributor is discussion prompted by mass media, in particular radio and posters. Radio is an extremely efficient medium in Rwanda, which has only one local language, nationwide broadcast and free public service announcements. Though radio was used to horrific effect in the 1994 genocide, its power has now been harnessed for good as most Rwandans listen to the radio and health information is disseminated quickly. Of critical importance is that couples’ testing, while strongly encouraged, is not mandatory. In comparison, Lusaka is home to Zambians from all five major language groups and 73 dialects. While Nyanja is the lingua franca, Zambians prefer to listen to radio stations broadcasting in their maternal language. There are many different stations, all expensive with none offering discounts for public service announcements. As a result few Zambians reported hearing about CVCT on the radio. One third of Rwandan CVCT clients reported having seen a CVCT poster, compared with only 3 % of their Zambia counterparts, which may reflect the significantly higher literacy rate in Kigali compared with Lusaka [31].

Our study had several limitations. RZHRG staff with differing levels of education and experience conducted the INA, INL and couple interviews. Though all interviewers were trained in questionnaire administration using standard training tools, differences in interviewing may have influenced results. Although all possible precautions were taken to maintain consistency, translations and back translations of questions may have impacted the understanding of questions. It is also possible that INAs and INLs over reported their promotional activities in the community.

INLs and INAs in this analysis included a combination of INLs from a pilot test of recruitment and training as well as INLs and INAs recruited early into subsequent promotional work. Therefore, INL and INA demographics reported previously differ slightly from those reported here -- namely a smaller percentage of Rwandan and Zambian INLs were men and a smaller percentage of Zambian INLs reported testing with their spouse relative to previous publications [19, 22].

## Conclusions

Findings from this study support the ability of social networks and influential community leaders to promote HIV prevention activities in their communities, specifically a tiered approach to reach couples via one-on-one and public or group promotional modalities. The study results suggest that recruitment of influential community leaders should prioritize collaboration with those who have previously tested for HIV so as to promote from a more informed and personal perspective. In terms of training, findings indicate the importance of public speaking and skills to target promotional activities toward couples, married men, and pre-marital boyfriends/girlfriends. Overall, this sustainable model could allow for an increase in utilization of CVCT services in these countries, and may have broader application to other sub-Saharan African countries to increase CVCT uptake leading to high-impact HIV prevention.

## Abbreviations

CVCT, couples’ voluntary counseling and testing; IAVI, international AIDS vaccine initiative; INAs, influential network agents; INLs, influential network leaders; IRB, Institutional Review Board; PSF, Project San Francisco; RZHRG, Rwanda Zambia HIV Research Group; SAS, statistical analysis software; USAID, United States Agency for International Development; VCT, voluntary HIV counseling and testing; ZEHRP, Zambia Emory HIV Research Project
